# Adjuvant Chemotherapy for Elderly Patients with Gastric Cancer after D2 Gastrectomy

**DOI:** 10.1371/journal.pone.0053149

**Published:** 2013-01-24

**Authors:** Ying Jin, Miao-zhen Qiu, De-shen Wang, Dong-sheng Zhang, Chao Ren, Long Bai, Hui-yan Luo, Zhi-qiang Wang, Feng-hua Wang, Yu-hong Li, Rui-hua Xu

**Affiliations:** 1 State Key Laboratory of Oncology in South China, Guangzhou, China; 2 Department of Medical Oncology, Sun Yat-sen University Cancer Center, Guangzhou, China; University of Porto, Portugal

## Abstract

**Background:**

A phase III clinical trial has already shown the survival benefits of postoperative chemotherapy in gastric cancer. However, there are limited published data concerning the elderly. This study aims to investigate the use of adjuvant chemotherapy for gastric cancer after D2 gastrectomy among the elderly and identify its impact on survival.

**Methods:**

We retrospectively reviewed 360 patients who had undergone D2 gastrectomy, aged 65 years or older, with non-metastatic gastric cancer in a single institution. We analyzed the predictors and survival benefits of adjuvant chemotherapy use in the elderly. Further, we analyzed the survival benefits of adjuvant chemotherapy by dividing the patients into groups according to disease stages and chemotherapeutic regimens.

**Results:**

Among the 360 patients, only 34.7% of patients received adjuvant chemotherapy. Age, tumor location, lymph node involvement and tumor invasion were associated with the receipt of adjuvant chemotherapy. Adjuvant chemotherapy improved the overall survival for non-metastatic elderly patients (HR 0.60, 95%CI 0.42–0.83, P = 0.003). Significant survival benefits were found with adjuvant chemotherapy in stage III patients (HR 0.67, 95%CI 0.47–0.97, P = 0.033), but not in stage I patients or in stage II patients (HR 0.52, 95%CI 0.21–1.30 P = 0.161). Compared to adjuvant chemotherapy without platinum, no significant survival benefits were observed with platinum-containing chemotherapy (HR 0.84, 95%CI 0.49–1.45, P = 0.530). Besides adjuvant chemotherapy, other independent prognostic factors of survival included tumor location, tumor size, histologic grade, depth of tumor invasion, and lymph node status.

**Conclusions:**

This study demonstrated the survival benefits of adjuvant fluoropyrimidine-based chemotherapy among the elderly patients with non-metastatic gastric cancer after D2 gastrectomy. However, due to the limitations of this study, further well-designed prospective studies with large populations are needed to confirm these findings and identify the patients that can tolerate and benefit from adjuvant chemotherapy.

## Introduction

As the second leading cause of cancer-related death worldwide, gastric cancer is a health threat. The only available treatment to cure gastric cancer is surgical resection, but the prognosis of the patients that have received radical resection is still poor. Therefore, adjuvant therapy is considered to improve the survival for patients with curative gastric resection. Several meta-analyses suggested that adjuvant therapy resulted in a small but significant advantage [1], [2], [3]. The U.S. Intergroup study(INT-0116) and the United Kingdom Medical Council MAGIC trial showed survival benefits from postoperative chemoradiotherapy and perioperative chemotherapy, respectively [4], [5]. However, most of the gastrectomy procedures in both studies were limited to D0 or D1 resections, while D2 resection only comprised about 10% of the cases. Therefore, the evidence cannot be generalized to patients in East Asia where D2 gastrectomy is the standard treatment. The recently reported five-year outcome of the Adjuvant Chemotherapy Trial of TS-1 for Gastric Cancer (ACTS-GC) study and the Capecitabine and Oxaliplatin Adjuvant Study in Stomach Cancer (CLASSIC) study confirmed the efficacy of the S-1 and XELOX regimen in adjuvant chemotherapy after D2 surgery compared with surgery alone [6], [7].

As the population continues to age, gastric cancer in the elderly will become an increasing clinical challenge. However, the elderly are less likely to receive the recommended treatment because of their shorter life expectancy, higher incidence of comorbidities, and higher risk of complications [8], [9]. Thus the effectiveness of postoperative chemotherapy for elderly patients (aged 65 or older) should be carefully assessed to avoid overtreatment or undertreatment. However, there are limited published data concerning the elderly. The elderly are underrepresented in clinical trials, and detailed clinicopathological data are limited.

This study aims to investigate the use of adjuvant chemotherapy among patients aged 65 years or older with non-metastatic gastric cancer after D2 gastrectomy in a single center and explore the survival benefits from chemotherapy.

## Materials and Methods

### Patients

We retrospectively analyzed the medical records of 360 patients who were pathologically proved and diagnosed with non-metastatic staged I through IV(M0) gastric adenocarcinoma according to American Joint Committee on Cancer (AJCC, seventh edition). All of the patients received curative gastrectomy with D2 nodal dissection by experienced surgeons in the Cancer Center of Sun Yat-Sen University between 1999 and 2007. We excluded 162 patients because of the presence of residual tumors or palliative surgery. There were 216 patients excluded because of distal metastasis, refusal of surgery, intolerance to surgery, death within 1 month of surgery, primary or secondary tumor history, neoadjuvant chemotherapy or adjuvant radiotherapy.

The adjuvant chemotherapy was mainly based on fluoropyrimidine, with or without a combination of other agents that included oxaliplatin, cisplatin, lobaplatin, paclitaxel, docetaxal, etoposide, doxorubicin, epirubicin, leucovorin, mitomycin. We recorded the cycles and regimens of chemotherapy of all patients in detail.

We retrospectively reviewed the clinicopathological features, including age at diagnosis, gender, comorbidities, tumor size, tumor location, depth of tumor invasion, number of retrieved lymph nodes, number of metastatic lymph nodes, histological grade, and tumor stage. We also collected the follow-up data of tumor recurrence or metastases and survival. The last follow-up data were collected prior to April 30^th^, 2012.

### Statistical analysis

All statistical analyses were performed using SPSS software, version 16.0. The clinicopathologic characteristics of the cohort were described, and the differences of these characteristics between the treatment groups were compared. Ordinal data were compared using the chi-square test, and continuous data were compared using a Mann-Whitney U test. A logistic regression model was created, using the treatment group as a dependent variable and potential clinicopathologic factors as covariables. Unadjusted Kaplan-Meier survival curves with log rank testing were generated to compare the survival benefits between treatment groups of all patients, and for each stage. The disease-specific survival would be analyzed by the Cox proportional hazards regression once the survival curves of the treatment groups separated. All potential predictors were taken into consideration for overall survival analysis. The hazard ratio and 95% confidence interval were used to estimate the role of each independent predictor of survival.

## Results

### Clinicopathologic characteristics

The median age of the 360 elderly patients with non-metastatic resectable gastric carcinoma was 69 years (range 65 to 83). The male to female ratio was 2.71∶1. Most patients in this study were more likely to have a Charlson comorbidity score [10] of 1 or less (96.1%). The patients were likely to have tumors in the proximal third of the stomach (60.6%), tumors smaller than 5 cm (60.3%), tumors that were poorly differentiated (65.0%), and tumors corresponding to AJCC stage II to III (33.1% and 55.8%, respectively). Among the 360 patients, 5.6% had T1 disease, 10.6% had T2 disease, 10.0% had T3 disease, and 73.9% had T4 disease. In this study, 22.5% of patients had N1 lymph node involvement, 20.0% had N2, and 18.1% had N3 (Table 1). Only 34.7% of patients (n = 125) received adjuvant chemotherapy, 6.9% (n = 25) received monochemotherapy and 27.8% (n = 100) received polychemotherapy. Among those 125 patients who received chemotherapy, 98.4% (n = 123) received fluoropyrimidine-based chemotherapy, 64.8% (n = 81) received chemotherapy included platinum, 9.6% (n = 12) received chemotherapy included paclitaxel or docetaxal, 5.6% (n = 7) received chemotherapy included etoposide, 4% (n = 5) received chemotherapy included doxorubicin or epirubicin, and 4% (n = 5) received chemotherapy included mitomycin. The number of the cycles ranged from 1 to 10, and there was a median of 3 cycles.

**Table 1:** Clinicopathological characteristics of patients aged 65 or older with non-metastatic resected gastric cancer.

Characteristic	Total	Surgery only	Surgery+chemotherapy	P
No. of patients	360	235	125	
Age at diagnosis				0.003*
65–70 years	189(52.5)	110(46.9)	79(63.2)	
>70 years	171(47.5)	125(53.1)	46(36.8)	
Gender				0.535
Male	263(73.1)	169(71.9)	94(75.2)	
Female	97(26.9)	66(28.1)	31(24.8)	
Charlson score				0.144
0	270(75)	171(72.7)	99(79.2)	
1	76(21.1)	52(22.1)	24(19.2)	
2+	14(3.9)	12(5.1)	2(1.6)	
Time of diagnosis				0.276
1999–2003	161(44.7)	110(46.8)	51(40.8)	
2004–2007	199(55.3)	125(53.2)	74(59.2)	
Location of tumor				<0.001*
Proximal	218(60.6)	165(70.2)	53(42.4)	
Distal	142(39.4)	70(29.8)	72(57.6)	
Size				0.862
≤5 cm	217(60.3)	144(61.3)	75(60.0)	
>5 cm	141(39.2)	91(38.7)	50(40.0)	
Grade				0.001*
Well or moderately differentiated	126(35.0)	96(40.9)	30(24.0)	
Poorly differentiated or undifferentiated	234(65.0)	139(59.1)	95(76.0)	
T category				0.012*
T1	20(5.6)	18(7.7)	2(1.6)	
T2	38(10.6)	27(11.5)	11(8.8)	
T3	36(10.0)	24(10.2)	12(9.6)	
T4	266(73.9)	166(70.6)	100(80.0)	
N category				<0.001*
N0	142(39.4)	116(49.4)	26(20.8)	
N1	81(22.5)	50(21.3)	31(24.8)	
N2	72(20.0)	42(17.9)	30(24.0)	
N3	65(18.1)	27(11.5)	38(30.4)	
AJCC stage(7th)				<0.001*
I	40(11.1)	34(14.5)	6(4.8)	
II	119(33.1)	91(38.7)	28(22.4)	
III	201(55.8)	110(46.8)	91(72.8)	

Abbreviations: CI, confidence interval; T, tumor; N, lymph node; AJCC, American Joint Committee on Cancer.

* Statistically significant.

### Predictors of receiving adjuvant chemotherapy

By univariate logistic regression analysis, age (reference age 65–70 years: age>70 years, odd ratio (OR) 0.51, 95% confidence interval (CI) 0.33–0.80, P = 0.003), tumor location (reference location proximal: distal, OR 3.20, 95% CI 2.04–5.03, P<0.001), depth of tumor invasion (reference depth of tumor invasion T1:T2, OR 3.67, 95%CI 0.73–18.54, P = 0.116; T3, OR 4.50, 95%CI 0.89–22.67, P = 0.068; T4, OR 5.42, 95%CI 1.23–23.86, P = 0.025), and lymph node involvement (reference lymph node status N0:N1, OR 2.77, 95%CI 1.49–5.13, P = 0.001; N2, OR 3.19, 95%CI 1.69–6.00, P<0.001; N3, OR 6.28, 95%CI 3.27–12.04, P<0.001) were associated with receiving adjuvant chemotherapy after D2 resection. The patient's gender (reference gender male: female, OR 0.84, 95% CI 0.51–1.39, P = 0.504), Charlson index (reference score 0: 1, OR 0.80, 95%CI 0.46–1.37, P = 0.414; 2+, OR 0.29, 95%CI 0.06–1.31, P = 0.108), tumor size (reference size ≤5 cm: >5 cm, OR 1.06, 95%CI 0.68–1.64, P = 0.813), and diagnosis time(reference time 1999–2003: 2004–2007, OR 1.28, 95%CI 0.82–1.98, P = 0.275) were not associated with the receipt of adjuvant therapy(Table 2). Age (reference age 65–70 years: age>70 years, adjusted OR 0.51, 95%CI 0.31–0.83, P = 0.007), tumor location (reference location proximal: distal, OR 3.96, 95% CI 2.40–6.58, P<0.001),lymph node involvement (reference lymph node status N0:N1, OR 2.36, 95%CI 1.22–4.57, P = 0.011; N2, OR 3.24, 95%CI 1.63–6.44, P = 0.001; N3, OR 5.19, 95%CI 2.56–10.50, P<0.001), and depth of tumor invasion (reference depth of tumor invasion T1:T2, OR 3.58, 95%CI 0.65–19.62, P = 0.142; T3, OR 5.12, 95%CI 0.91–28.67, P = 0.063; T4, OR 5.29, 95%CI 1.11–25.27, P = 0.037) were still associated with the receipt of adjuvant therapy by multivariate logistic regression analysis (Table 3).

**Table 2:** Univariate predictors of receiving adjuvant chemotherapy among the 360 patients aged 65 or older with non-metastatic resected gastric cancer.

Covariate	Odd Ratio	95% Cl	P
Age at diagnosis			0.007*
65–70 years	1.00	Referent	-
>70 years	0.51	0.33–0.80	0.003*
Gender			
Male	1.00	Referent	-
Female	0.84	0.51–1.39	0.504
Time of diagnosis			
1999–2003	1.00	Referent	-
2004–2007	1.28	0.82–1.98	0.275
Chalson index			
0	1.00	Referent	-
1	0.80	0.46–1.37	0.414
2+	0.29	0.06–1.31	0.108
Location of tumor			
Proximal	1.00	Referent	-
Distal	3.20	2.04–5.03	<0.001*
Size			
≤5 cm	1.00	Referent	-
>5 cm	1.06	0.68–1.64	0.813
Grade			
Well or moderately differentiated	1.00	Referent	-
Poorly differentiated or undifferentiated	2.19	1.35–3.56	0.002*
T category			
T1	1.00	Referent	-
T2	3.67	0.73–18.54	0.116
T3	4.50	0.89–22.67	0.068
T4	5.42	1.23–23.86	0.025*
N category			
N0	1.00	Referent	-
N1	2.77	1.49–5.13	0.001*
N2	3.19	1.69–6.00	<0.001*
N3	6.28	3.27–12.04	<0.001*
AJCC stage			
I	1.00	Referent	-
II	1.74	0.66–4.58	0.259
III	4.69	1.89–11.66	0.001*

Abbreviations: CI, confidence interval; T, tumor; N, lymph node; AJCC, American Joint Committee on Cancer.

* Statistically significant.

**Table 3:** Factors associated with the receipt of adjuvant chemotherapy by multivariate analysis for patients aged 65 or older with non-metastatic resected gastric cancer.

Covariate	Adjusted Odd Ratio	95% Cl	P
Age at diagnosis			
65–70 years	1.00	Referent	-
>70 years	0.51	0.31–0.83	0.007*
Location of tumor			
Proximal	1.00	Referent	-
Distal	3.96	2.40–6.58	<0.001*
T category			
T1	1.00	Referent	-
T2	3.58	0.65–19.62	0.142
T3	5.12	0.91–28.67	0.063
T4	5.29	1.11–25.27	0.037*
N category			
N0	1.00	Referent	-
N1	2.36	1.22–4.57	0.011*
N2	3.24	1.63–6.44	0.001*
N3	5.19	2.56–10.50	<0.001*

Abbreviations: CI, confidence interval; T, tumor; N, lymph node.

* Statistically significant.

### Survival analysis

**Figure 1:**
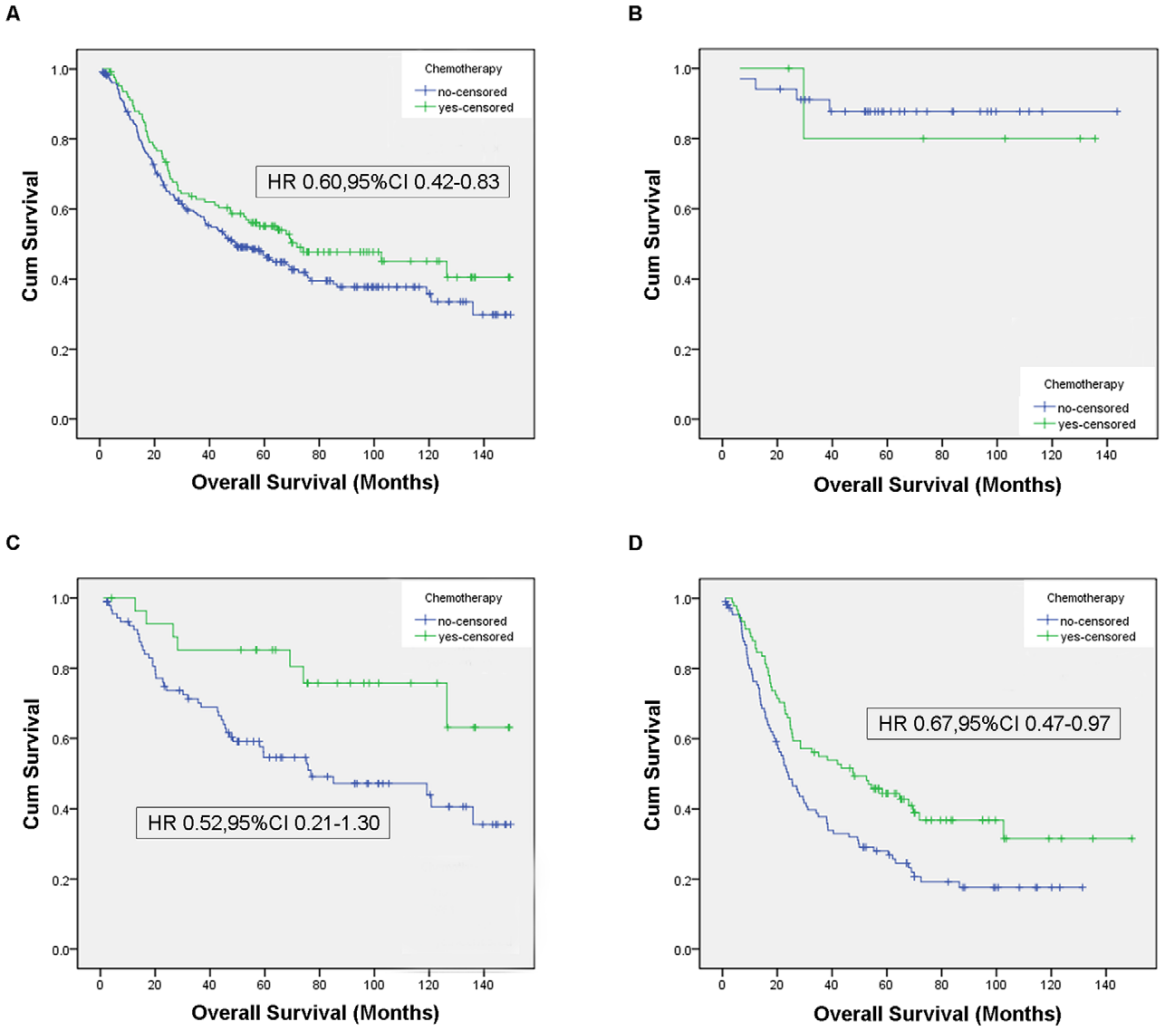
Unadjusted Kaplan-Meier curves, overall and by stages. Figure 1A shows the unadjusted Kaplan-Meier curves of the 360 elderly patients. Figure 1B shows the unadjusted Kaplan-Meier curves of the elderly patients with stage I gastric adenocarcinoma. Figure 1C shows the unadjusted Kaplan-Meier curves of the elderly patients with stage II gastric adenocarcinoma. Figure 1D shows the unadjusted Kaplan-Meier curves of the elderly patients with stage III gastric adenocarcinoma. The Hazard ratio were generated from a multivariate Cox regression model adjusted for age, gender, Chalson index, tumor site, histologic grade, size, and treatment group.

**Figure 2:**
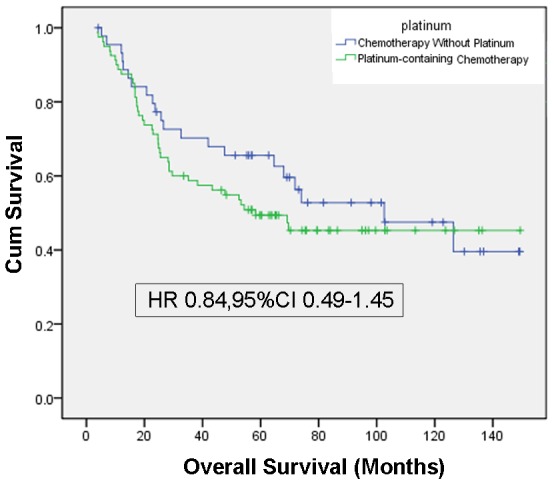
Unadjusted Kaplan-Meier curves, by chemotherapeutic regimens. Figure 2 shows the unadjusted Kaplan-Meier curves of the elderly patients who received platinum-containing chemotherapy and chemotherapy without platinum. The Hazard ratio were generated from a multivariate Cox regression model adjusted for age, gender, Chalson index, tumor site, histologic grade, size, depth of tumor invasion, and lymph node involvement.

During the follow-up period, 195(55%) patient died from tumors, and 6 died from other diseases. The median followed-up was 46.7 months after gastectomy (range 1.1 to 149.3). Unadjusted Kaplan-Meier survival curves were constructed for all patients, and for each stage (Figure 1). The median survival in this study was 55.9 months among the patient who received surgery plus adjuvant chemotherapy and 39.6 months among the patients who received surgery alone (P = 0.083, Figure 1A). The median months of survival for surgery alone group vs. surgery/adjuvant chemotherapy group was 57.4 months vs. 88.0 months for stage I (P = 0.72, Figure 1B), 49.9 vs. 77.6 for stage II (P = 0.026, Figure 1C), 22.4 vs. 46.5 for stage III (P = 0.004, Figure 1D). The median months of survival for platinum-containing chemotherapy group vs. chemotherapy without platinum group was 53.2 months vs. 62.8 months (P = 0.314, Figure 2).

Using Cox proportional hazards multivariate analysis, proximal tumor (P = 0.001), larger tumor (P = 0.004), higher histologic grade (P = 0.004), deeper tumor invasion (P = 0.001), and lymph node involvement (P<0.001) were negative independent prognostic factors. On multivariate analysis, adjuvant chemotherapy was associated with a significantly reduced risk of death (HR 0.60, 95%CI 0.42–0.83, P = 0.003) among this elderly population (Table 4). Significant survival benefits were found with adjuvant chemotherapy in stage III patients (HR 0.67, 95%CI 0.47–0.97, P = 0.033), but not in stage I patients or in stage II patients (HR 0.52, 95%CI 0.21–1.30 P = 0.161)(Figure 1). Compared to adjuvant chemotherapy without platinum, no significant survival benefits were observed with platinum-containing chemotherapy (HR 0.84, 95%CI 0.49–1.45)(Figure 2)

**Table 4:** Multivariate prognostic factors for overall mortality among the 360 patients with non-metastatic resected gastric cancer.

Variate	HR	95% Cl	P
Age at diagnosis			
65–70 years	1.00	Referent	-
>70 years	1.22	0.91–1.62	0.19
Gender			
Male	1.00	Referent	-
Female	0.87	0.62–1.22	0.421
Chalson index			
0	1.00	Referent	-
1	1.12	0.79–1.58	0.521
2+	0.68	0.28–1.69	0.409
Time of diagnosis			
1999–2003	1.00	Referent	-
2004–2007	0.98	0.72–1.33	0.886
Location of tumor			
Proximal	1.00	Referent	-
Distal	0.55	0.39–0.77	0.001*
Size			
≤5 cm	1.00	Referent	-
>5 cm	1.54	1.14–2.07	0.004*
Grade			
Well or moderately differentiated	1.00	Referent	-
Poorly differentiated or undifferentiated	1.64	1.18–2.30	0.004*
T category			
T1/T2	1.00	Referent	-
T3	1.65	0.75–3.65	0.217
T4	2.90	1.54–5.45	0.001*
N category			
N0	1.00	Referent	-
N1	2.03	1.34–3.07	0.001*
N2	2.22	1.46–3.37	<0.001*
N3	3.49	2.25–5.39	<0.001*
Treatment			
Surgery alone	1.00	Referent	-
Adjuvant chemotherapy	0.60	0.42–0.83	0.003*

Abbreviations: CI, confidence interval; T, tumor; N, lymph node.

* Statistically significant.

## Discussion

Although the 5-year outcome of a randomized phase III ACTS-GC study showed that postoperative adjuvant treatment could improve overall survival in patients with gastric cancer who had undergone D2 gastrectomy, the subgroup analysis indicated that the survival benefits decrease as patient age increases [7]. Moreover, there were no statistically significant effects of postoperative chemotherapy for patients aged older than 70(HR 0.779, 95%CI 0.527–1.151). In the CLASSIC study, for which the collection of the overall survival data are not yet completed, the subgroup analysis of the 3-year disease-free survival showed more benefits of chemotherapy for patients 65 or older [6]. It was reported that disease-free survival was strongly correlated with overall survival based on the GASTRIC data. Therefore, the delivery of adjuvant chemotherapy to elderly patients with gastric cancer after D2 gastrectomy remains a dilemma for physicians.

In this single center study, we examined the clinicopathological characteristics associated with delivery of adjuvant chemotherapy to 360 elderly patients with non-metastatic gastric cancer after D2 gastrectomy, providing insight into the current treatment recommendations for the elderly in China. We also evaluated the efficacy of delivering adjuvant chemotherapy to elderly patients in China and observed that patients had a significant survival benefit from postoperative chemotherapy in this study cohort.

In our study, only approximately one third of the patients received postoperative chemotherapy. Within this population, the patients aged older than 70 were less likely to receive chemotherapy compared to younger patients. This finding is similar to several other cancers, including prostate cancer, ovarian cancer, breast cancer and colorectal cancer [11], [12], [13], [14], where elderly patients are less likely to receive adjuvant therapy even if the treatments are known to be effective and tolerable. There may be various reasons of this result. Older patients may have more comorbid diseases, they might be less tolerant to chemotherapy, and they may prefer to undergo less treatment in their relatively limited lifetime [15], [16]. In the CLASSIC study, 56% of patients who received the XELOX chemotherapy regimen experienced grade 3 or 4 adverse events, such as neutropenia, thrombocytopenia, vomiting, nausea [6]. In the ACT-GC study, 22.8% of patients with monochemotherapy experienced grade 3 or 4 adverse events [7]. The possible treatment toxicity may be an important barrier for the elderly to receive adjuvant therapy, especially when the patient has a decrease in physical status due to gastectomy.

Hoffman et al. [17]analyzed 1,023 elderly patients with resected gastric cancer in the linked Surveillance, Epidemiology, and End Result-Medicare database (SEER), and they reported that patients diagnosed during the later months of the study were more likely to receive adjuvant chemoradiation therapy. This was mainly due to the report of the INT-0116 trial in 2000. Strauss et al. reported similar results in a study including 1,993 elderly patients with non-metastatic gastric adenocarcinoma. However, the diagnosis time did not significantly influence the administration of adjuvant chemotherapy to patients during the study period in our study. The possible explanation may be that chemotherapy was not considered as a standard or recommended adjuvant treatment until the 3-year result of ACTS-GC study published in 2007.

It was reported in a previous study of the elderly that having fewer comorbidities was an independent factor associated with receipt of adjuvant treatment [17], [18]. However, most of the patients in our study had a Charlson comorbidity score of 0 or 1, because they were required to be able to tolerate the surgery. Thus, the comorbidity score was not a statistically significant predictor of receiving chemotherapy in our study cohort. The size, location, and histologic grade of the tumor are potential prognostic factors of the survival of resectable gastric cancer, so that they are associated with the use of adjuvant chemotherapy. However, the prognostic importance of these parameters is inconsistent in different studies [18], [19], [20], influencing the predictive value of receipt of adjuvant chemotherapy. In our study, the location and histologic grade of the tumor was significantly associated with the receipt of adjuvant chemotherapy of the elderly.

In our study cohort, we found that adjuvant chemotherapy significantly improved overall survival for non-metastatic elderly patients with a median survival of 16.3 months longer (HR 0.60, 95%CI 0.42–0.83). In subgroup analysis stratified by disease stages, treatment was associated with an HR for tumor-specific death of 0.67 and a median survival that was 24.1 months longer compared to surgery alone for stage III patients. For stage II patients, there was no significant benefit for group that received the surgery plus postoperative chemotherapy. However, the P value of the log-rank test for stage II patients between treatment groups was 0.026, and the survival curves of two treatment groups were separate. What's more, the patients with stage II disease trended toward survival improvement with adjuvant chemotherapy with an HR smaller than 1 and a longer median survival. We suggest that this result may be caused by the small proportion of patients (n = 28) who received chemotherapy with stage II disease. Similarly, the analysis of the role of adjuvant chemotherapy in stage I disease in this cohort was limited by small numbers of patients. The survival benefits of adjuvant chemotherapy reported based on the data of the phase III trial ACTS-GC study were similar (HR 0.669, CI 0.540–0.828) [7]. Several meta-analyses also reported that chemotherapy could reduce the risk of death in gastric cancer after curative resection (HR ranged from 0.72 to 0.90) [1], [3], [21], [22]. After evaluating the results from 20 randomized clinical trials, Mari, et al. reported that chemotherapy reduced the risk of death by 18% (HR 0.82, 95% CI 0.75–0.89) [1]. Liu, et al. evaluated 19 qualified clinical randomized trials and reporded that adjuvant chemotherapy could improve the survival rate after curative resection(RR 0.85, 95%CI 0.80–0.90) [22]. Paoletti, et al. recently published the result of a meta-analysis of survival data from 17 trials involving 3,838 patients and demonstrated that adjuvant chemotherapy was associated with a statistically significant benefit for overall survival (HR 0.82, 95% CI 0.76∼0.90) [2]. Similar studies with opposite conclusion also reported recently. Hoffman et al. reported that elderly patients may not gain a survival benefit from the administration of adjuvant chemoradiation after analyzing elderly patients with resected gastric cancer in the SEER-Medicare database [17]. But most of the gastrectomy procedures in these studies were limited to D0 or D1 resections, and the postoperative treatment is adjuvant chemoradiation. Dittmar et al. recently reported that there was a trend toward longer survival for the elderly patients who underwent gastric resection plus chemotherapy [23]. However, the gastric resection in this retrospective study included both radical and palliative resection.

So far, there is no standard regimen for adjuvant chemotherapy. Some suggested that patients would benefit from adjuvant chemotherapy and should not be influenced by different schemes, including monotherapy, double therapy and triple therapy [1], [22]. Some also reported that statistically significant benefits could be detected from a fluoropyrimidine-based monochemotherapy regimen and a fluoropyrimidine-based polychemotherapy regimen but not from chemotherapy regimens without fluoropyrimidines [2]. In our study, 123 out of 125(98.4%) patients received fluoropyrimidine-based monochemotherapy or polychemotherapy. We examined the monochemotherapy group and the polychemotherapy group versus surgery alone and only detected a significant survival benefit of polychemotherapy over surgery alone. The absence of a survival benefit in monochemotherapy group may be due to the small proportion of patients (n = 25) that received monochemotherapy. We examined the overall survival between the patients received platinum-containing chemotherapy and chemotherapy without platinum, and did not detect significant survival differences between groups. The number of patients who received chemotherapy included taxane (n = 12), etoposide (n = 7), anthracyclines (n = 5), or mitomycin (n = 5) was small to undergo further analysis. We suggested that elderly patients with resected gastric cancer may gain a survival benefit from the fluoropyrimidine-based chemotherapy, with or without platinum.

In addition to adjuvant chemotherapy, there were other independent negative predictors of survival for the elderly, including a more proximal site, larger tumor, poorer differentiation, deeper tumor invasion, and lymph node involvement. These prognostic factors have been reported in previous contexts as well [20], [24].

Our study has various limitations. First, this study is based on retrospective data. However, the bias may be reduced by the fact that these data were collected from a single institution. Second, the patients received various chemotherapy regimens as new regimens were developed during the period of this study. Thus, we cannot conclude a specific regimen recommended from this study. Third, the proportion of patients with early disease who received chemotherapy or those who received monochemotherapy was small, which makes it difficult to stratify the patients for further analysis.

In this retrospective, single institution study, we demonstrated the survival benefits of adjuvant fluoropyrimidine-based chemotherapy present among the elderly with non-metastatic gastric cancer after D2 gastrectomy. Further well-designed prospective studies with larger populations are needed to confirm these findings. Elderly patients are highly variable in their functional status, reserve capacity, and comorbidity. Thus further studies are needed to identify the patients that can tolerate and gain benefit from adjuvant chemotherapy.
